# TOM1L Is Involved in a Novel Signaling Pathway Important for the IL-2 Production in Jurkat T Cells Stimulated by CD3/CD28 CoLigation

**DOI:** 10.1155/2009/416298

**Published:** 2010-02-21

**Authors:** Ahmed Elmarghani, Hanan Abuabaid, Peter Kjellen

**Affiliations:** ^1^School of Science and Technology, Orebro University, 70182 Orebro, Sweden; ^2^Molecular Cell and Biology Laboratory, The Salk Institute for Biological Studies, La Jolla, CA 92037, USA

## Abstract

TOM1L (target of Myb-1 Like) was identified as a binding partner for the full length and catalytically-active Lck in a yeast 2-hybrid screening assay. Here we show that in Jurkat T cells stimulated by CD3/CD28 coligation where the expression of TOM1L is reduced by lenti virus mediated-siRNA results in a dramatically lower IL-2 production. The production of IL-2 in siRNA treated cells stimulated with PMA/ionomycin was not affected indicating an involvement of TOM1L in a pathway proximal of TCR and CD28. The coexpression of Fyn with TOM1L increased the level of the phosphorylated form of Fyn indicating that TOM1L has the ability to activate Fyn. The ability of TOM1L to activate Fyn was further shown in a kinase assay using angiotensin II as a substrate. By confocal microscopy, we show that the expression of TOM1L in non-treated HeLa and SK-N-SH cells colocalizes with the mitochondrial membrane but not with lysosomal compartments or the trans-Golgi network. Furthermore, we show that the over-expression of TOM1L in Jurkat cells causes an increase of the STAT3 expression . Based on our results, we here propose that TOM1L is involved in a novel signaling pathway that is important for the IL-2 production in T cells.

## 1. Introduction

The function and property of the TOM1L (target of Myb-1 Like) gene are to a large extent unknown. TOM1L is a paralog of TOM1 [1] and belongs to a new family of proteins containing an N-terminally located VHS (Vps27p/Hrs/Stam) domain and a central GAT (GGA (Golgi-localizing, *γ*-adaptin ear domain homology, ADP-ribosylation factor (Arf)-binding protein and Tom) domain. The GAT domain of both TOM1L and Tom1 has the ability to bind ubiquitin and Tollip (Toll-interacting protein), whereas the C-terminal regions of the Tom1-family proteins interact with clathrin [2, 3]. Tollip is a negative regulator of signaling downstream of the IL-1 receptor and the Toll-like receptors [4, 5]. Inhibition by Tollip is mediated through its ability to suppress the activity of the IL-1 receptor associated kinase (IRAK). When TOM1L-proteins are coexpressed with Tollip, they are associated with the endosomes to where they recruit clathrin [3]. The interaction of TOM1L-proteins with ubiquitin and clathrin suggests a functional role in sorting proteins via multivesicular bodies (MVBs) to lysosomes and subsequent degradation. Alternatively, the proteins are recycled to the trans-Golgi network (TGN) or the plasma membrane. Mass spectrometry has been used to identify proteins which were specifically tyrosine-phosphorylated, or complexed with a tyrosine-phosphorylated protein, as a result of fibroblast growth factor receptor-1 (FGFR-1) overexpression in human 293T cells [6]. Among the identified proteins was Tom1L, which has not previously been described in FGFR signaling and was proposed to be a new docking molecule in FGFR-1 signaling [6].

The kinase activity of Src-family kinases is dramatically increased by the direct binding of proteins to the SH3-domain that destabilizes the intramolecular structure of Src-family kinases [7, 8]. Thus, it is therefore of great importance to identify proteins, which contain binding domains for the SH3 domain and determine whether they can regulate the kinase activity. Most SH2 and/or SH3 binders increase Src-family activity in vivo and exhibit mitogenic and/or transforming activity [9, 10].

Activation of the Src-family kinases Lck and Fyn is central to the initiation of TCR signaling pathways [11]. One of the first signaling events in activation of T-helper cells (T_h_) by antigen presenting cells (APCs) is that Lck becomes phosphorylated and thereby activated. How Lck is activated during TCR engagement has not been fully defined. TCR-cross-linking alone does not stimulate Lck autophosphorylation and CD45, which is an important phosphatase regulating the activity of Src-family kinases and signaling in lymphocytes [12], is not required for this process [13]. The CD4 molecule recruits Lck to the T-cell/APC interface, whereas CD28 is suggested to sustain Lck activation [13].

The mouse homolog of TOM1L, Srcasm (Src activating and signaling molecule), has been shown to interact with Fyn in a yeast two-hybrid screen of a murine keratinocyte library [14]. Srcasm serves as a substrate for Fyn and activates Fyn kinase and is also capable of interacting with Grb2 and the regulatory subunit of phosphoinositide 3-kinase, p85, in a phosphorylation-dependent manner [14].

## 2. Materials and Methods

### 2.1. Cell Lines

A human leukemic T-cell line, Jurkat, that expresses the large T antigen of SV40 virus was grown in RPMI 1640 medium supplemented with 10% fetal calf serum, 2 mM glutamine, and penicillin/streptomycin. Human kidney cells, 293T, HeLa SK-N-SH cells were grown in DMEM supplemented with 10% bovine calf serum and penicillin/streptomycin.

### 2.2. Tom1L Antibodies and Immunohistochemistry

The fragment of the far C-terminal end of the TOM1L gene encoding amino acids 243–476 was cloned in pGEX-KG (Amersham Pharmacia Biotech). The TOM1L-GST fusion proteins were expressed in BL21 or XL1-Blue *Escherichia coli*. GST fusion proteins bound to glutathione Sepharose 4B (Amersham Pharmacia Biotech) were eluted in 50 mM Tris–HCl pH 8.0 containing 5 mM reduced glutathione (Sigma, MO). All DNA constructs were verified by DNA sequence analysis. To produce anti-TOM1L antibodies, rabbits were immunized with an emulgate containing equal amounts of pTOM1L-GST and mineral oil. At different time points after immunization, the rabbits were bled and serum was isolated. Immune histochemistry performed using confocal fluorescence microscopy. Cells were placed on to collagen type-I coated cover slips and fixed with 3.7% paraformaldehyde in PBS containing 2% sucrose. The cells were incubated with the anti-TOM1L antibodies for 2 hours at room temperature washed with PBS. This was followed by incubation with the Alexa Fluor488 (green-fluorescent) or Alexa Fluor568 (red-fluorescent) conjugated secondary antibody (no. A11034, no. A11036 Molecular Probes, Invitrogen, Carlsbad, CA) for 1 h at room temperature. MitoTrackerRed (no. M7512 Molecular Probes, Invitrogen, Carlsbad, CA) was used to visualize the mitochondria according to the procedure recommended by the manufacturer.

### 2.3. Transfection, Western Blotting and Immunoprecipitation

To prepare full length TOM1L, DNA for transfection was RNA isolated from Jurkat T cells (RNeasy easy kit, Qiagen) and used for subsequent cDNA synthesis (Amersham biotech) followed by PCR. TOM1L was cloned into pCS3 + MT to append six Myc-tags to the amino terminus. Transient transfection was performed as described earlier [15]. 293T cells were seeded at 3 × 10^5^ cells per 6 cm gelatin-coated Petri dishes the day prior to transfection. Forty-eight hours after transfection with Lck (pCEP4), Fyn (pCS3) alone or together with Myc-tagged TOM1L were cells resuspended in 1 mL of lysis buffer (1% Nonidet-P40, 20 mM Tris pH 8.4, 150 mM sodium chloride, 2 mM EDTA, and 200 *μ*M sodium vanadate). Lysates were clarified by centrifugation at 34,000 g for 30 minutes at 4°C. After preclearing with Protein-A Pansorbin cells (Calbiochem, CA), the lysates were incubated for 30–40 minutes at 4°C with 1 *μ*L of the specified primary antiserum followed by a second 60-minute incubation with Protein A Pansorbin cells. Immunoprecipitates were washed two to three times in lysis buffer. Immunoprecipitates and cell lysates were subjected to SDS–PAGE and transferred to Immobilon-P (Millipore, MA) for Western blotting (Bio-Rad, CA). Immobilon-P filters were blocked by incubation in rinse buffer (10 mM Tris pH 7.4, 150 mM NaCl, and 0.1% NaN3), supplemented with 3% bovine serum albumin and then stained with monoclonal anti-Myc antibody, rabbit anti-Lck antibodies, or rabbit anti-phosphotyrosine antibodies. Bound antibodies were detected with ^125^I–protein A (Amersham Pharmacia Biotech, NJ) and the Typhoon 8600 Variable Mode Imager (Molecular Dynamics, CA). For siRNA experiments, the cells were lysed in lysis buffer (1% NP-40, 20 mM Tris 7.6 pH, 150 mM NaCl, 5 mM EDTA at 2 × 10^7^ cell/mL supplemented with phosphatase inhibitors) (phosphatase proteases inhibitor (no. SC 24948, Santa Cruz, CA)). The cell lysates were subjected to SDS–PAGE and transferred to nitrocellulose membrane (High bond ECL, no. 613-3555, Amersham) for Western blotting (Bio-Rad, CA). The anti-TOM1L antibody was detected by goat-anti rabbit IgG conjugated with alkaline phosphatase (S3731, Promega, WI) and Western Blue Stabilized Substrate (S3841, Promega, WI).

### 2.4. Yeast 2-Hybrid Assay

Competent AH109 yeast cells of *Saccharomyces cerevisiae *(Clontech, Palo Alto, CA, USA) containing four reporter genes (*ADE2, HIS3, lacZ and MEL1*), under the control of three distinct GAL4-responsive promoters were used in a yeast 2-hybrid assay. A human thymus cDNA library (Human Thymus MATCHMAKER cDNA Library, pACT-2 vector, Clontech) with about 3 × 10^6^ independent clones containing the activation domain (AD) of the GAL4 was used as prey. The bait was Lck cloned in the pGBT9 plasmid (Clontech) containing the binding domain (BD). Selected AD containing plasmids clones was amplified and the nucleotide sequences were determined using pACT-2 specific primers.

### 2.5. Lentivirus and siRNA

Lentivirus was harvested by centrifugation of the supernatant from 293T cells cotransfected with the third generation of HIV-based vectors with a conditional packaging system [16, 17]. Two 64-mere oligonucleotides (5′GATCCCCATCCAACTTACCTTGTCACTTGAAGAGAGTGACAAGGTAAGTTGGATTTTTTGGAAA3′) forming a hairpin containing the DNA-sequence for siRNA and restriction enzyme sequences were annealed and cloned into pSUPER SK(+) having the H1-RNA promoter. This sequence including the H1-RNA promoter was then subcloned into the transfer vector cPPT-GFP (p156RRLsinPPTCMVGFPPRE) using EcoR I/Kpn I. 293T cells were transfected with 10 *μ*g of cPPT-GFP (containing the siRNA construct or empty), 6.5 *μ*g of pRRE, 2.5 *μ*g of pRev, and 3.5 *μ*g of pVSV-G vectors (kind gift from Inder Verma lab, Salk inst., La Jolla) using the Lipofectamine 2000 system (Invitrogen, CA).

### 2.6. ELISA

Supernatants of Jurkat cells (2.5 × 10^5^ cells/well, 96-well TC plate) were harvested after 48 hours of stimulation with plate bound anti-CD3 (MAB100) and soluble anti-CD28 (MAB342) -antibodies (R and D Systems, Minneapolis, MN). Amount of IL-2 in the supernatants was quantified according the ELISA-kit provided by eBiosciences, La Jolla, CA. Alternatively were Jurkat cells stimulated with 50 ng/mL of PMA plus 0.5 *μ*g/mL of ionomycin for 48 hours. Data represents one out of three experiments performed in triplicates. The Student *T*-test was used for statistical analysis.

### 2.7. In Vitro Kinase Assay

Immunoprecipitates were incubated with 5 *μ*Ci of (*γ*-32P) ATP (Amersham Pharmacia Biotech) and 2 mM (Val5)-angiotensin II (Sigma) as an exogenous substrate in 1,4-piperazinediethanesulfonic acid (PIPES) kinase buffer (40 mM PIPES pH 7.0, 10 mM MnCl_2_) for 1, 3, and 5 minutes at room temperature. The reactions were stopped at the indicated times by adding 5% trichloroacetic acid and centrifuged at 10,000 *g *for 10 minutes at 4°C. Supernatants were subsequently absorbed onto P81 phosphocellulose paper (Whatman, U.K.). The phosphocellulose paper was washed with 0.5% phosphoric acid to remove unincorporated (*γ*-32P) ATP. Incorporated *γ*-32P was measured using liquid scintillation counting.

### 2.8. Luciferase Assay with the STAT3 Reporter

T-antigen Jurkat cells were transfected with 0.6 *μ*g of firefly luciferase reporter STAT3 (Signal-transducer-andactivator-of-transcription-3) and 0.6 *μ*g of each experimental plasmid DNA. Transfections were performed with the lipofectamine 2000 (Invitrogen). Included in each transfection was 25 ng of a reporter construct that expresses *Renilla *luciferase under the control of the *β*-actin promoter. The luciferase assay was performed using the Promega Dual-Luciferase Reporter system (Promega, WI). 48 hours after the transfection, approximately 2 × 10^6^ cells were washed in cold PBS and lysed in 100 *μ*L of lysis buffer (Promega) for 10 minutes at 4°C. Twenty *μ*L from each lysate was mixed with 100 *μ*L of assay buffer (Promega) immediately prior to reading in a Berthold Lumat LB 9507 luminometer (Perkin–Elmer, MD). The luciferase assay was quenched by adding 100 *μ*L of Stop and Glo (Promega). The activity of *Renilla *luciferase in each sample was then immediately measured and used to normalize the luciferase data.

### 2.9. Statistical Analysis

The data are presented with means and SEM. The Student *T*-test was used to analyze the data. A *P*-value < .05 was considered statistically significant. (*) = *P*-value < .05 and (**) = *P*-value < .01.

## 3. Results

### 3.1. TOM1L and Fyn Activity

**Figure 1:**
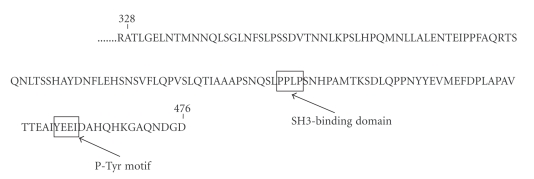
Amino acid sequence of the TOM1L clone that was picked up seven times in the yeast 2-hybrid assay. The SH3-binding domain (LPPLP) and the phosphotyrosine motif (P-Tyr) at the C-terminus of TOM1L are marked.

**Figure 2:**
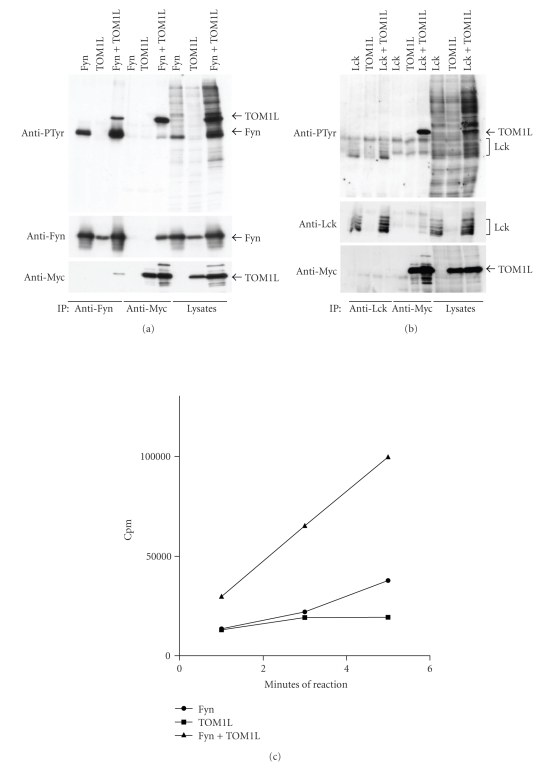
(a) 293T cells transfected with plasmids encoding Fyn alone or together with Myc-tagged full length TOM1L. Fyn and TOM1L proteins were isolated by immunoprecipitation using anti-Fyn, or anti-Myc antibodies and assayed by SDS−PAGE and immunoblotting as described in material and methods. Immunoblots were analyzed with primary anti-phosphotyrosine (anti-PTyr), anti-Fyn or anti-Myc –antibodies, respectively. (b) 293T cells transfected with plasmids encoding Lck alone or together with Myc-tagged full length TOM1L. Lck and TOM1L proteins were isolated by immunoprecipitation using anti-Lck or anti-Myc antibodies and assayed by SDS−PAGE and immunoblotting as described in material and methods. (c) Kinase assay: Anti-Fyn immunoprecipitates were incubated with 5 *μ*Ci of (*γ*-32P) ATP and 2 mM (Val5)-angiotensin II as an exogenous substrate in PIPES kinase buffer for 1, 3, and 5 minutes at room temperature.

We identified TOM1L as a binding partner to Lck in a yeast 2-hybrid screening assay using full length catalytically-active Lck as bait. Seven clones from a human thymus cDNA library containing DNA sequences of TOM1L were picked up as binding partners to Lck (Figure 1). For this reason was TOM1L chosen for further functional studies. The TOM1L clone contains an SH-3 binding domain sequence at amino acids 421–425 (LPPLP) and a P-Tyr motif (YEEI) at amino acids 460–463 (Figure 1). 293T cells were transfected transiently with plasmids encoding TOM1L alone or TOM1L together with Lck or Fyn in order to examine the role of TOM1L on the activity of Lck and Fyn. In lysates from 293T cells where TOM1L was coexpressed with Fyn (Figure 2(a)) or Lck (Figure 2(b)), the amount of phosphorylated proteins (anti-PTyr staining) increased considerably, indicating that the overexpression of TOM1L causes an overall increased kinase activity in these cells. Coexpression of Fyn or Lck with TOM1L increased the level of the phosphorylated form (anti-PTyr staining) of anti-Fyn immunoprecipitated Fyn (Figure 2(a)) and anti-Lck immunoprecipitated Lck (Figure 2(b)). This indicates that TOM1L has the ability to activate both Fyn and Lck, although the increase of anti-PTyr staining of the anti-Fyn immunoprecipitate was much stronger compared with the anti-Lck immunoprecipitate. The level of staining using anti-Fyn antibodies in anti-Fyn immunoprecipitates from cells transfected with TOM1L alone represents the endogenous level of Fyn (Figure 2(a)). Endogenous levels of Lck in anti-Lck immunoprecipitates from cells transfected with TOM1L alone were not detectable (Figure 2(b)).

TOM1L was considerably phosphorylated in anti-Myc immunoprecipitated lysates, where TOM1L was cotransfected with Fyn (Figure 2(a)) or Lck (Figure 2(b)), indicating that TOM1L is a substrate for both Fyn and Lck. 

The ability of TOM1L to activate Fyn was further examined in a kinase assay using the substrate angiotensin II (Figure 2(c)). The ability of Fyn to phosphorylate angiotensin II in a 1-, 3- or 5- minute reaction increased considerably in anti-Fyn antibody precipitates of lysates where Fyn was cotransfected with TOM1L compared with anti-Fyn antibody precipitates of lysates where Fyn was transfected with an empty vector (Figure 2(c)).

### 3.2. Intracellular Localization of TOM1L

In order to examine the intracellular localization of TOM1L, a rabbit anti-TOM1L antibody was made and immune histochemistry was performed using confocal fluorescence microscopy. The SK-N-SH neuroblastoma and HeLa cell lines were used for the immune staining. 

**Figure 3:**
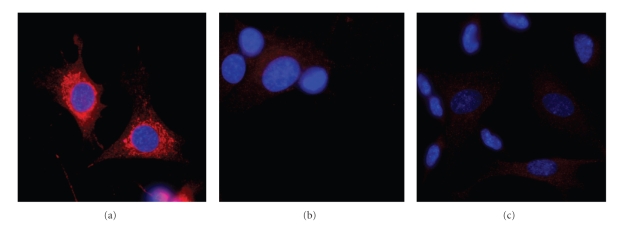
Cellular expression of TOM1L in SK-N-SH neuroblastoma cells examined by confocal microscopy. Confocal imaging of cells stained with antibodies against TOM1L (red) (a), normal rabbit serum (b) or antibodies against TOM1L together with recombinant TOM1L (c). Cells were stained with DAPI (blue) in all images to visualize the localization of the nucleus.

The intracellular expression of TOM1L was examined by staining SK-N-SH neuroblastoma cells with the rabbit anti-TOM1L antibody (Figure 3(a) **)**. To examine the specificity of the anti-TOM1L antibody, the same cells were stained with normal rabbit serum (Figure 3(b)) or antibodies against TOM1L together with recombinant TOM1L protein (Figure 3(c)). We concluded that the anti-TOM1L antibody specifically stained on organelle around the nucleus.

**Figure 4:**
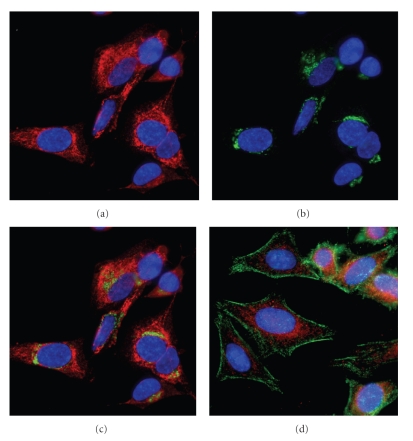
Confocal imaging of HeLa cells stained with antibodies against TOM1L (red) and anti-trans-Golgi network. Individual images were separated digitally: (a) anti-TOM1L (red), (b) anti-trans-Golgi network (green), and merged images (c). (d) HeLa cells stained with anti-TOM1L (red) and anti-Cathepsin D antibodies (green). Cells were stained with DAPI (blue) to visualize the localization of the nucleus.

**Figure 5:**
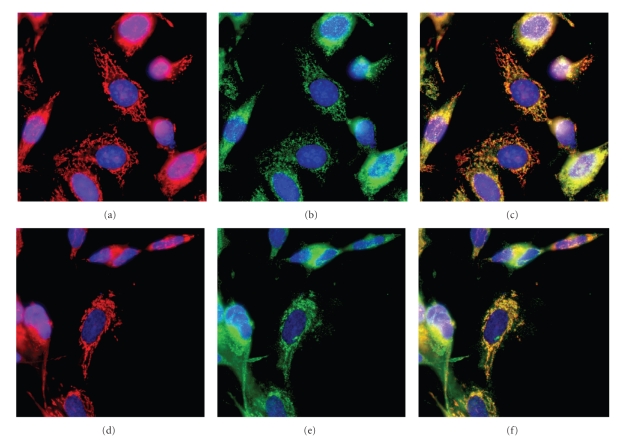
Cellular expression of TOM1L colocalize with the mitochondria. Confocal imaging of SK-N-SH neuroblastoma cells ((a)–(c)) or HeLa cells ((d)–(f)) stained with MitoTracker Red and antibodies against TOM1L. Individual images were separated digitally: ((a) and (d)) MitoTracker Red (red), ((b) and (e)) anti-TOM1L (green), and merged images ((c) and (f)). Cells were stained with DAPI (blue) to visualize the localization of the nucleus.

To identify which organelle that was stained by the anti-TOM1L antibody, we used the anti-TGN38 for the trans-Golgi network (Figures 4(a)-4(b)) and the anti-Cathepsin D antibodies for lysomal compartments (Figure 4(d)). None of these colocalized with anti-TOM1L antibody staining, however, we found a good match when merging the MitoTracker Red staining with the anti-TOM1L antibody staining, as shown in Figure 5(c) (SK-N-SH cells) and Figure 5(f) (HeLa cells). 

#### 3.2.1. Reduction of the TOM1L Expression by siRNA and IL-2 Production

**Figure 6:**
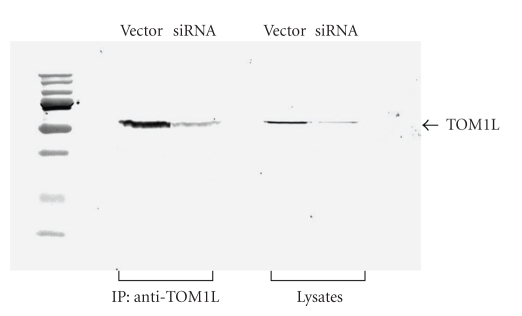
The protein expression of TOM1L in whole lysates and immunoprecipitated lysates (anti-TOM1L antibodies) examined by western blot analysis in Jurkat cells after siRNA treatment. The siRNA experiments were performed by lenti virus-mediated infection of a hairpin construct. The expression of TOM1L was efficiently reduced in cells infected with the hairpin construct (siRNA) as compared with cells infected with a vector lacking the hairpin construct (Vector).

**Figure 7:**
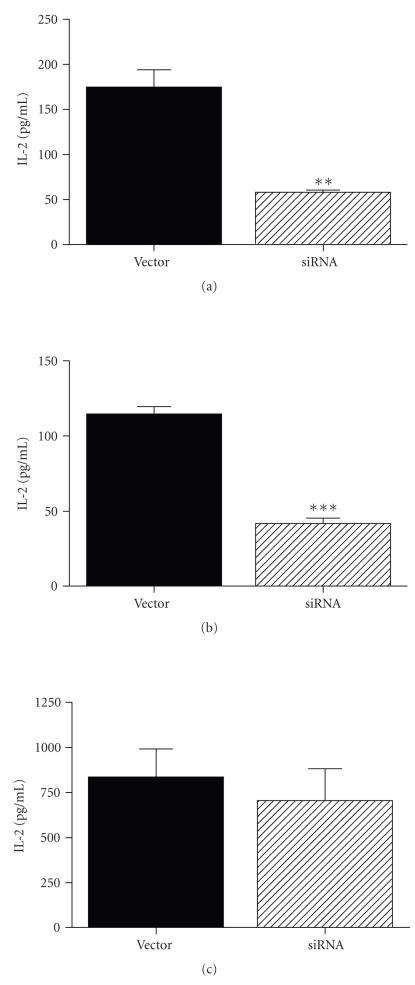
Il-2 production of stimulated Jurkat cells where the expression of TOM1L was reduced by siRNA. The IL-2 production was significantly lower in stimulated cells infected with the hairpin construct (siRNA) as compared with cells infected with a vector lacking the hairpin construct (Vector). (a) Stimulation with plate bound anti-CD3 (1 *μ*g/well) plus 1 *μ*g/mL of anti-CD28 antibodies, 48 hours, (b) stimulation with plate bound anti-CD3 (1 *μ*g/well) plus 0.25 *μ*g/mL of anti-CD28 antibodies, 48 hours, (c) cells stimulated with 50 ng/mL of PMA plus 0.5 *μ*g/mL of ionomycin for 48 hours.

Since Fyn and Lck are predominately expressed in T-cells, we performed siRNA experiments using Jurkat T-cells to further investigate the functional role of TOM1L. The siRNA experiments were performed by lenti virus-mediated infection of a hairpin construct. The expression of TOM1L was efficiently suppressed in Jurkat cells infected with the hairpin construct as compared with cells infected with a vector lacking the hairpin construct (negative control) (Figure 6). To examine the role of TOM1L in effector T-cell activation, the Jurkat cells were stimulated for IL-2 production by plate bound anti-CD3 antibodies and soluble anti-CD28 antibodies. We found that the level of IL-2 production was significantly lower in siRNA-infected cells compared with negative control (Figures 7(a) and 7(b)). This effect could not be seen in the same siRNA-infected cells after stimulation with PMA plus ionomycin (Figure 7(c)), indicating that the function of TOM1L is downstream and proximal of TCR and CD28.

### 3.3. Over-Expression of TOM1L and STAT3

**Figure 8:**
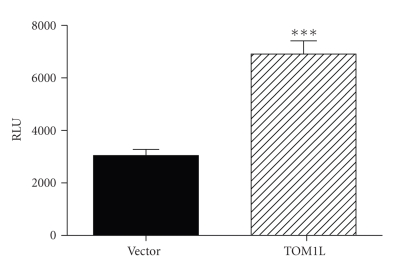
Jurkat cells were transfected with a firefly luciferase reporter for STAT3 (Signal-transducer-andactivator-of-transcription-3) together with plasmids encoding TOM1L or the empty plasmid construct. Included in each transfection was a reporter construct that expresses *Renilla *luciferase under the control of the *β*-actin promoter. 48 hours after the transfection, the cells were washed and lysed followed by reading in a luminometer. The activity of *Renilla *luciferase in each sample was used to normalize the luciferase data.

Jurkat cells were transfected with plasmids encoding the STAT3 (Signal-transducer-andactivator-of-transcription-3) firefly luciferase reporter and TOM1L. The over-expression of TOM1L in T cells caused a significantly increased output from the STAT3 gene reporter (Figure 8).

## 4. Discussion

The function of TOM1L in T cells has not been described earlier; however, the function as a negative regulator of mitogenesis in epithelial cells stimulated with growth factors has been proposed [18, 19]. It has been proposed that Tom1L1 negatively regulates Src mitogenic signaling induced by platelet-derived growth factor (PDGF) through modulation of Src-receptor association [18]. Further has it proposed that upon association with CHC (clathrin heavy chain) will Tom1L1 reduce the level of Src in caveolae, thereby preventing its association with the PDGF receptor, which is required for the induction of mitogenesis [19]. Our results suggest a mitogenic role of TOM1L in T cells, indicated by the dramatically reduced IL-2 production in cells where the expression of TOM1L was reduced by siRNA. A mitogenic function of TOM1L in T cells was further indicated by the increased expression level of STAT3 after overexpression of TOM1L. STAT3 is known to be a substrate for SFK members [20], and SFK inhibition in various carcinoma tumor cell lines results in loss of STAT3 activity [21]. STAT3 is closely linked with tumorigenesis and its role in cancer is indicated by numerous avenues of evidence, including that STAT3 regulates the expression of genes that mediate proliferation (e.g., c-myc and cyclin D1), suppress apoptosis (e.g., Bcl-xL and survivin), or promote angiogenesis (e.g., VEGF) (reviewed in [22]). In a comprehensive genomic and expression profiling study, of chromosome 17 in breast cancer cells, TOM1L was recently identified as a novel candidate gene [23]. The contrasting results about the mitogenic effect of TOM1L imply that the function of TOM1L is dependent on the cell type and the way the cells are stimulated.

Reorganization of proteins around the T-cell/APC interface, termed immunological synapse (IS) formation, plays an important role in the initial steps and sustained activation of T-cells [13]. Very little is known about organelle redistribution during IS formation in T_h_ cells, however and very interestingly, it recently has been shown that T_h_-cell activation requires mitochondrial translocation to the immunological synapse [24]. In this study, the authors show that the redistribution of the mitochondria to the IS was necessary to maintain Ca^2+^ influx across the plasma membrane and Ca^2+^-dependent T-cell activation. In the present study, our immune histochemistry data suggest that the TOM1L protein is localized to the mitochondrial membrane in resting and nontransfected cells. The translocation of the mitochondria to the IS during T cell activation could hypothetically serve as explanation for how TOM1L could interact with Fyn/Lck in vivo. This mechanism would be possible for TOM1L since Lck/Fyn are bound to the plasma membrane and proximal to the TCR and CD28 [11, 25].

## 5. Conclusions

Taken together, our results imply that TOM1L has a mitogenic effect in Jurkat T-cells and is involved in a novel cell signaling pathway that is crucial for the IL-2 production and STAT3 expression. Understanding the mechanisms that control the activity of the Lck/Fyn in T cells is of great importance for understanding the progression of cancer and autoimmune disease.
